# Intra-tumoral susceptibility signals in brain gliomas: where do we stand?

**DOI:** 10.3389/fradi.2025.1546069

**Published:** 2025-02-20

**Authors:** Simone Cataldi, Paola Feraco, Maurizio Marrale, Pierpaolo Alongi, Laura Geraci, Ludovico La Grutta, Giuseppe Caruso, Tommaso Vincenzo Bartolotta, Massimo Midiri, Cesare Gagliardo

**Affiliations:** ^1^ Department of Biomedicine, Neurosciences and Advanced Diagnostics (BIND), University of Palermo, Palermo, Italy; ^2^ Centre for Medical Sciences (CISMed), University of Trento, Trento, Italy; ^3^ Department of Physics and Chemistry “Emilio Segrè”, University of Palermo, Palermo, Italy; ^4^ Nuclear Medicine Unit, Department of Radiological Sciences, A.R.N.A.S. Civico, Palermo, Italy; ^5^ Neuroradiology Unit, Department of Radiological Sciences, A.R.N.A.S. Civico, Palermo, Italy; ^6^ Department of Health Promotion, Mother and Child Care, Internal Medicine and Medical Specialties (ProMISE), University of Palermo, Palermo, Italy; ^7^ Neuroradiology Unit, University-Hospital Paolo Giaccone, Palermo, Italy

**Keywords:** magnetic resonance imaging, susceptibility-weighted imaging, intra-tumoral susceptibility signals, brain, glioma

## Abstract

Nowadays, the genetic and biomolecular profile of neoplasms—related with their biological behaviour—have become a key issue in oncology, as they influence many aspects of both diagnosis and treatment. In the neuro-oncology field, neuroradiological research has recently explored the potential of non-invasively predicting the molecular phenotype of primary brain neoplasms, particularly gliomas, based on magnetic resonance imaging (MRI), using both conventional and advanced imaging techniques. Among these, diffusion-weighted imaging (DWI), perfusion-weighted imaging (PWI), MR spectroscopy (MRS) and susceptibility-weighted imaging (SWI) and have been used to explore various aspects of glioma biology, including predicting treatment response and understanding treatment-related changes during follow-up imaging. Recently, intratumoral susceptibility signals (ITSSs)—visible on SWI—have been recognised as an important new imaging tool in the evaluation of brain gliomas, as they offer a fast and simple non-invasive window into their microenvironment. These intratumoral hypointensities reflect critical pathological features such as microhemorrhages, calcifications, necrosis and vascularization. Therefore, ITSSs can provide neuroradiologists with more biological information for glioma differential diagnosis, grading and subtype differentiation, providing significant clinical support in prognosis assessment, therapeutic management and treatment response evaluation. This review summarizes recent advances in ITSS applications in glioma assessment, emphasizing both its potential and limitations while referencing key studies in the field.

## 1 Introduction

Genetic and biomolecular analyses of neoplasms have become increasingly critical in the era of precision and personalized medicine, as they reflect biologic behaviour of tumours. Consequently, biomolecular analyses have increasingly complemented traditional pathological assessments for both diagnosis and management in oncology. This principle is equally relevant in neuro-oncology, as reflected in the last editions (2016 and 2021) of World Health Organization (WHO) classification of central nervous system (CNS) tumors, which have progressively emphasized the role of genetic and biomolecular data in brain gliomas evaluation. For example, the newest classification of diffuse gliomas—the most common primary malignant brain neoplasms—is primary based on specific genetic alterations, such as isocitrate dehydrogenase (IDH) genes mutations and 1p/19q codeletion (1–3).

Currently, diagnosis and characterization of gliomas require histopathological analysis of a sample obtained through surgical biopsy. Therefore, neuroradiologists play a critical role in identifying imaging features—primarily through magnetic resonance imaging (MRI) with both conventional and advanced sequences—that may suggest the underlying nature of a primary brain tumor before biomolecular and pathological analysis (4, 5).

Over the last decades, Susceptibility-Weighted Imaging (SWI) has emerged as improved substitute of conventional T2*-weighted Gradient Echo (GRE) imaging and has become a standard component of MRI protocols (6, 7). SWI exploits magnetic susceptibility differences in brain tissues, enabling visualization of paramagnetic and diamagnetic substances such as calcium, blood products and iron.

This review explores the role of intratumoral susceptibility signal (ITSS), an imaging parameter defined as low-signal-intensity fine-linear or dot-like structures, with or without conglomeration, visible within tumors on SWI. These ITSSs are variably associated with intratumoral microhemorrhage, necrosis, vascular proliferation, and calcifications, providing a unique insight into glioma biology (8).

## 2 The 2016 and 2021 WHO classifications of CNS tumors: brief simplified story of an epochal change

The 2016 and the latter 2021 WHO Classification of CNS tumors marked a significant shift by integrating molecular profiling into the characterization of gliomas. This updated framework moves beyond traditional histopathological evaluation to incorporate genetic and molecular alterations, providing a more precise and clinically relevant understanding of gliomas (1–3). Nowadays the most important molecular markers are isocitrate dehydrogenase (IDH) mutation status, 1p/19q co-deletion, genetic alterations in the promoter region of the telomerase reverse transcriptase (TERT) gene, O6-Methylguanine-DNA Methyltransferase (MGMT) promoter methylation status, Ki-67 nuclear protein proliferation index and X-linked alpha thalassaemia intellectual disability syndrome (ATRX) gene mutations. All these play pivotal roles in defining glioma subtypes and their prognostic implications.

For example, gliomas are now classified into IDH-mutant or IDH-wildtype categories, with IDH mutation generally associated with better outcomes (9–11). Additionally, the identification of 1p/19q co-deletion distinguishes oligodendrogliomas from astrocytomas, further refining diagnosis and guiding therapeutic decisions (12).

TERT promoter mutations are found predominantly in IDH-wildtype glioblastomas and oligodendrogliomas with 1p/19q co-deletion, while they are rare in IDH-mutant astrocytomas, highlighting their diagnostic relevance in differentiating glioma subtypes (13–15).

MGMT is a DNA repair enzyme that removes alkyl groups from the O6 position of guanine, a site commonly damaged by alkylating chemotherapeutic agents like temozolomide (TMZ) so gliomas with a methylated MGMT promoter, the reduced repair capability increases the efficacy of alkylating agents, making chemotherapy more effective (16–19).

Ki-67 is a nuclear protein assessed through immunohistochemistry that is widely used as a marker of cellular proliferation in cancer diagnostics, including in brain gliomas (20). It is expressed during active phases of the cell cycle (G1, S, G2, and mitosis) but is absent in quiescent cells (G0). A high Ki-67 proliferation index indicates rapid cell division and aggressive tumor behavior, which is common in high-grade gliomas like glioblastomas while lower Ki-67 indices are typically associated with less aggressive, low-grade gliomas (21).

Mutations or loss of ATRX function are commonly observed in IDH-mutant gliomas and are associated with a specific alternative lengthening of telomeres (ALT) mechanism. This allows tumor cells to maintain telomere length independently of telomerase activation, enabling continued cell division (22–24).

## 3 Technical consideration for SWI: understanding the basis of ITSS

SWI, a high-resolution 3D GRE sequence, visualizes brain tissues on the basis of differences in their magnetic susceptibility. This susceptibility is an intrinsic magnetic property of substances and determines their response to an external magnetic field: substances with negative susceptibility, such as calcium (i.e., diamagnetic substances), oppose the applied field, while those with positive susceptibility, such as blood, iron, and deoxyhemoglobin (i.e., paramagnetic substances), enhance the field (6, 25).

SWI images are generated by separately acquiring magnitude and phase images. The magnitude images, combined with a phase mask derived from phase data, enhance the visibility of both diamagnetic and paramagnetic substances, appearing as areas of signal loss. An important consideration is how vendors present phase information on SWI phase images. In a left-handed system (i.e., Canon and Siemens) phase increases positively in a clockwise direction. Conversely, in a right-handed system (i.e., the more common configuration used on GE Healthcare, Philips and United Neusoft), phase increases positively in a counterclockwise direction-aligned with the way the fingers of your right-hand curl when forming a fist. Thus, choice between left-handed and right-handed systems affects the appearance of the resulting images. In clinical practice phase data are particularly useful for differentiating between calcium and blood: in calcifications, phase images exhibit low contrast in a left-handed system, while blood products show low contrast in a right-handed system. However, in routine practice, distinguishing microbleeds from microcalcifications can be challenging and sometimes inconclusive, particularly in larger or more geometrically complex lesions, which may produce intricate signal patterns on phase images (6, 25).

**Figure 1:**
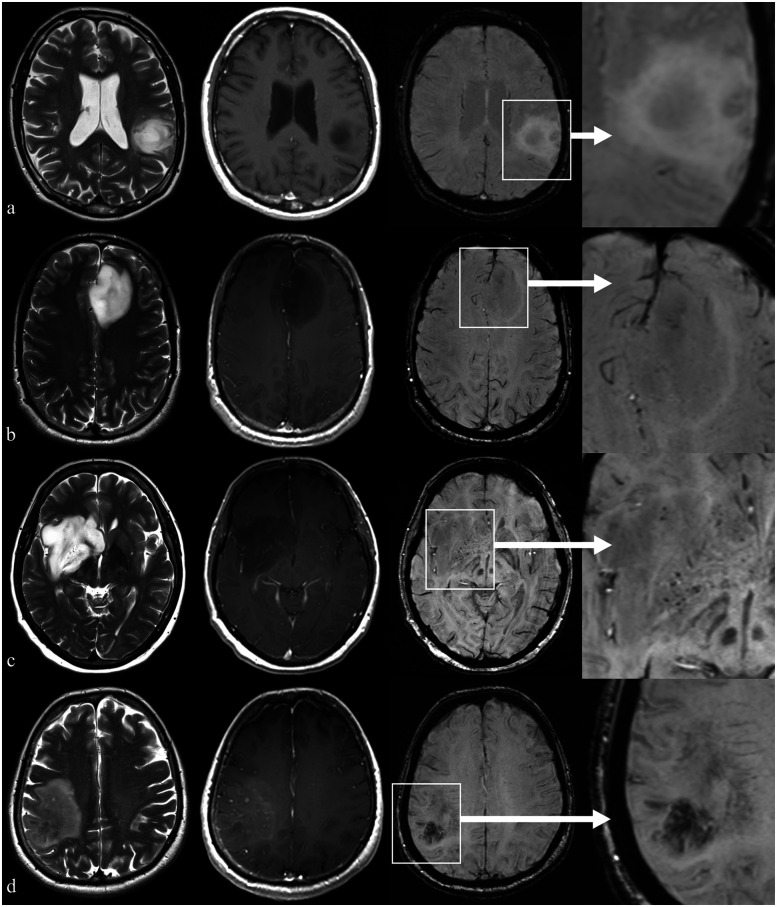
Example of patients with brain glioma presenting with different ITSS grades but similar findings on conventional imaging. First column: axial T2-weighted images; second column: axial T1-weighted images acquired after i.v. injection of gadolinium-based contrast medium; third column: axial 3D susceptibility-weighted images (SWI); last column: a magnified view of the area of interest on SWI. Row **(a)** parieto-temporal glioma infiltrating the left supramarginal and angular gyri in a 47 y.o. right-handed male showing no relevant findings on SWI (ITSS grade 0); row **(b)** left frontal glioma infiltrating the anterior cingulate gyrus in a 36 y.o. male with a cluster of (<5) tiny hypointense black dots in SWI (ITSS grade 1); row **(c)** right basal ganglia and insular glioma in a 55 y.o. female showing multiple (<10) millimetric intralesional hypointense spots on SWI (ITSS grade 2); row **(d)** right parietal glioma glioma in a 72 y.o. male showing diffuse hypointense subcortical deposits on SWI (ITSS grade 3).

In glioma microenvironment (Figure 1), ITSSs correspond to both microcalcifications and microhemorrhages or necrosis (visible as dot-like hypointensities), as well as to aberrant vascular proliferation (visible as fine-linear hypointensities) (8).

High-grade gliomas (HGGs) exhibit more prominent ITSSs due to their aggressive nature. These tumors invade fragile and abnormal blood vessels, leading to microbleeds, as well as in regions of micro- and macro-necrosis (26).

While rare in other glioma subtypes, intratumoral calcifications, better detected on SWI compared to GRE, are more common in IDH mutant, 1p/19q codeleted astrocytomas (oligodendrogliomas) (27, 28). In some cases, phase images can help distinguish between calcifications and microbleeds, offering a valid tool in differentiation of this subtype of glioma (25). Other potential causes of intratumoral calcification include products of previous intratumoral bleeding or treatment-related changes (29).

The first attempt to grade ITSS in gliomas was proposed by Park et al. which proposed a semi-quantitative approach consisting of counting ITSSs on the slice where they are most conspicuous: grade 0, no ITSS; grade 1, 1–5 ITSSs; grade 2, 6–10 ITSSs; and grade 3, ≥11 ITSSs (Table 1) (8).

**Table 1:** ITSS grading scale (0–3) according to Park et al. (8).

ITSS grade	Number of dot-like and/or fine linear ITSS	Tumor microenvironnement
0	None	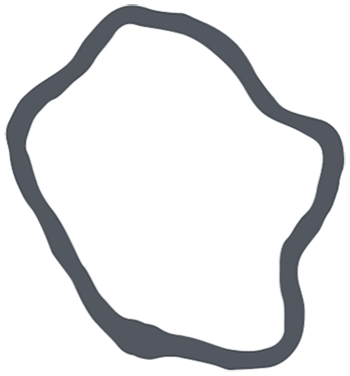
1	1–5	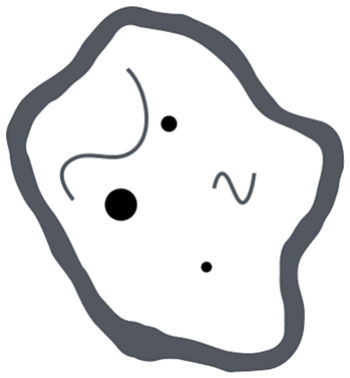
2	6–10	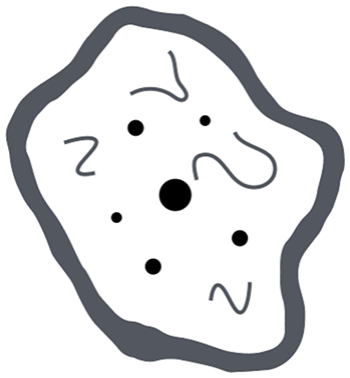
3	≥11	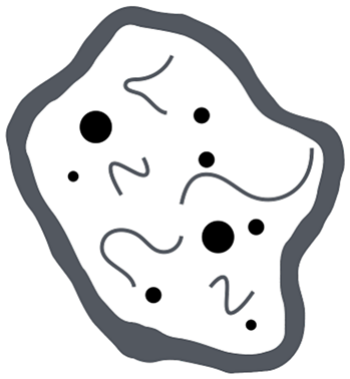

In the tumor microenvironment column black dots-like elements correspond to both microcalcifications and microhemorrhages or necrosis while fine black lines to aberrant vascular proliferation. All tumor microenvironment drawings included in this table were hand-drawn by Dr. Prof. Cesare Gagliardo.

## 4 The role of ITSS in differential diagnosis, grading, and molecular profiling of brain gliomas

Several studies have highlighted the potential of SWI as an accurate tool for differentiating solitary expansive brain lesions (30, 31). Particularly, HGGs exhibit a higher grade of ITSSs compared to primary CNS lymphoma (PCNSL) (30, 32, 33) and other non-neoplastic lesions (e.g., brain abscesses or tumefactive demyelinating lesions). Metastases demonstrate intermediate ITSSs grades, lower than HGGs but higher than those of PCNSL (34–36).

Focusing on gliomas, the number and patterns of ITSSs vary according to tumor grade. HGGs exhibit more prominent vascular structures (fine-linear ITSSs, often with a conglomerated pattern), microhemorrhages (dot-like structures) and areas of necrosis, compared to low-grade gliomas (LGGs), reflecting the more aggressive biological behaviour of these tumors (37–40). Hori et al. showed that the ratio between total ITSSs volume and tumor volume differentiates HGGs (grades 3 and 4) from LGGs (grades 1 and 2) (41). Yang et al. applied this approach to IDH-mutant astrocytomas, finding a greater number of ITSSs in grade 4 IDH-mutant astrocytomas compared to grade 2 and/or 3 (42). Bhattacharjee et al. attempt to discriminate between vascular and micro hemorrhagic components of ITSSs using R2* values. They found that the ITSS vasculature volume (IVV) could significantly differentiate not only HGGs from LGGs but also distinguish between different degrees of malignancy (grades 2 vs. 3, 2 vs. 4, and 3 vs. 4) (43). Thus, in adult patients, the quantity and heterogeneity of ITSSs increase with tumor grade, allowing differentiation between LGGs (with minimal dot-like and sparse linear ITSS) and HGGs (characterized by abundant ITSSs, often with a conglomerated pattern). However, this relationship is less consistent in the pediatric population. Gaudino et al. demonstrated that the absence of ITSSs often correlated with low-grade tumors in children. Conversely, the presence of ITSSs was not always indicative of high-grade tumors due to the extreme histological heterogeneity of pediatric brain tumors (44). Kong et al. demonstrated that ITSS levels are significantly higher in HGGs compared to LGGs. Furthermore, gliomas with IDH1 mutations exhibit lower ITSS grades than their IDH wild-type counterparts. Additionally, MGMT-methylated gliomas display lower ITSS grades than MGMT-unmethylated ones. However, no significant differences in ITSS grade were observed based on 1p/19q co-deletion status in the same study (45). Moreover, higher ITSS levels correlate with an elevated Ki-67 labeling index (Ki-67 LI) and ATRX gene wild-type status, both of which are markers of poorer prognosis in IDH-mutant astrocytomas (46).

### 4.1 Therapeutic management and follow-up of brain gliomas: assessing the feasibility of ITSS

As mentioned earlier, ITSS correlates with several biomolecular markers, providing a valuable prognostic imaging tool in glioma evaluation.

Highly vascularised gliomas are more responsive to antiangiogenic drugs compared to those with less vascularization or those that are necrotic (47). Thus, high levels of ITSSs—reflecting tumor aggressiveness and vascularity—may help in assessing the potential response to chemotherapy or radiotherapy in gliomas, in combination with other advanced MRI techniques such as DWI and PWI (48).

Lupo et al. demonstrated that the percentage of hypointense signals within contrast-enhancing lesions on susceptibility-weighted imaging can predict response to a treatment regimen combining anti-angiogenic therapy (enzastaurin), cytotoxic agents (temozolamide), and radiation therapy in gliomas (49).

Additionally, evaluating ITSS during follow-up imaging may provide valuable insights into therapeutic effects of chemotherapy and radiotherapy. A reduction in ITSS can signal a positive response, while an increase in ITSS may indicate tumor recurrence or progression, as well as treatment-related changes such as necrosis and calcifications. Furthermore, ITSS evaluation helps monitor vascular structural changes in glioblastomas, tracking the effects of anti-angiogenic therapy, cytotoxic chemotherapy, and radiation therapy *in vivo*, making it an effective tool for neuroradiological follow-up, as indicated by Grabner et al. (50).

However, Martucci et al. reported that, while the angiogenic profile of primary glioblastomas, measured using perfusion weighted imaging, may serve as an MRI biomarker for regorafenib response in recurrent glioblastomas but no significant correlation was observed between ITSS grade and regorafenib response was reported (51).

Recently, even neurosurgical research has explored the potential role of ITSS in planning brain tumor biopsies. Specifically, a significant correlation has been observed between high ITSS grades (particularly grade 3) and the risk of significant hemorrhage following stereotactic biopsy (STB) (52, 53).

Okamoto et al. suggested that ITSS grade (in combination with tumor volume) influences intraoperative blood loss during the surgical resection of pediatric posterior-fossa tumors (54). These findings support the routine use of SWI in pre-biopsy planning to perform safer biopsy techniques (open or endoscopic) and more accurate hemostasis in tumors with higher ITSS grades. Additionally, when multiple candidate biopsy sites are available, prioritizing areas with lower ITSS grades may offer a safer approach.

### 4.2 Emerging SWI-based MRI techniques for ITSS quantification

Nowadays, ITSS has become a well-recognized biomarker in neuro-oncology, playing a crucial role for glioma evaluation. However, several challenges remain in its application in routine clinical practice, primarily the lack of a standardized quantification method.

The semi-quantitative approach introduced by Park et al. was the first attempt to standardize ITSS evaluation. This method involves manually counting ITSSs on the slice where they are most conspicuous (8). While this approach is straightforward and easy to use, it has notable limitations, including a lack of comprehensive visualization of the tumor and significant operator dependency, which implicates subjectivity to the measurements.

To include comprehensive evaluation of the tumor, Hori et al. proposed a grading system based on the ratio of total SWI hypointense volume to overall tumor volume (41).

Radbruch et al. aimed to reduce operator dependence by proposing a percentage-wise quantification method for ITSS using automated post-processing techniques. This method has proven particularly valuable in differentiating between specific types of brain metastases (55, 56).

Moreover, Quantitative Susceptibility Mapping (QSM) has emerged as a promising tool to overcome the limitations of semi-quantitative methods. By providing quantitative measures of magnetic susceptibility, QSM eliminates operator dependency and can differentiate between distinct sources of ITSS, such as hemorrhage, calcification, and other susceptibility effects (25, 55, 57, 58).

As mentioned earlier, Bhattacharjee et al. proposed a quantitative approach that calculates the ITSS vasculature volume (IVV) within tumors. This technique leverages R2* values to filter out hemorrhagic contributions, offering a more precise evaluation of intratumoral microvasculature (43).

Recently, the fractal dimension (FD), introduced by Di Ieva et al., represents another innovative parameter derived from computational fractal-based analyses. Initially developed on a 7 T MRI scanner, this technique provides detailed reconstructions of the geometric architecture of SWI hypointensities. The FD offers an “architectural fingerprint” of gliomas, useful for both diagnostic and follow-up purposes (59, 60). Indeed, higher FD values correlate with higher glioma grades, reflecting increased complexity and vascular density. Furthermore, FD may be used to assess therapeutic effects, particularly in antiangiogenic therapies, by indicating favorable effects (such as decreased intratumoral microvasculature) or unfavorable results (such as increased microvasculature) (60).

### 4.3 Limitations, challenges, and future direction

ITSS patterns in gliomas necessitate careful interpretation, as the low signal intensity, particularly in dot-shaped lesions, may be attributable to microhemorrhages or calcifications on conventional magnitude images.

SWI parameters, including field strength and sequence settings, directly influence ITSS visibility as magnetic field strength increases, the number of identified ITSSs consequently rises. Therefore, it is recommended to use the same MRI system for follow-up studies, particularly for the same patients, to ensure consistency and reliability of results.

Protocol standardization is crucial, and the development of standardized or automated methods is necessary to improve the reproducibility of ITSS assessment. In this regard, artificial intelligence (AI)-based approaches, particularly deep learning algorithms for image acquisition, are being integrated into nearly all new MRI systems, helping reduce acquisition times and improve image quality (61, 62).

Longitudinal multicenter studies conducted with equipment from different vendors but with homogeneous protocols are essential to validate ITSS as a biomarker for glioma progression and treatment response. Advances in imaging technologies, such as ultra-high-field MRI, may further enhance ITSS detection. Although, on the other hand, the possibility of identifying ITSS even with scanners operating at 1.5 T, routinely used in the clinical setting outside of research and academic scenarios, makes this topic worthy of further research in differential diagnosis, grading, and molecular profiling of brain gliomas. Moreover, the implementation of ITSS paves the way for the possibility of a non-contrast brain tumor imaging protocol for patients who either have contraindications to contrast agents or are unable to tolerate bolus injections (8).

Additionally, integrating ITSS analysis with molecular and genetic profiling will significantly improve personalized management strategies for glioma patients especially if we consider modern and emerging new theranostics approaches like for instance transcranial focused ultrasound that offers non-invasive way to target tumors and enhancing drug delivery by modulating the blood-brain barrier (63).

Furthermore, nuclear medicine, combined with the evaluation of Intra-Tumoral Susceptibility Signals (ITSS), could significantly advance the diagnosis and treatment of brain gliomas. Nuclear imaging modalities such as positron emission tomography (PET) and single-photon emission computed tomography (SPECT) can provide functional and metabolic insights into tumor behavior, while ITSS analysis offers detailed structural and vascular information. Integrating these approaches could enhance tumor characterization, enabling precise localization, grading, and differentiation of gliomas. Furthermore, radiotheranostics could leverage ITSS data to optimize targeted delivery of therapeutic isotopes, maximizing treatment efficacy and minimizing damage to healthy tissue. This synergy holds promise for a more personalized and effective approach to managing brain gliomas (64).

Finally, calling into question a very hot topic in recent years, radiomics and artificial intelligence (AI) could revolutionize tumor diagnosis by enhancing the evaluation of Intra-Tumoral Susceptibility Signals (ITSS). Radiomics extracts quantitative features from medical images, such as geometrical features, textures, and intensities, that may not be visible to the human eye, enabling a deeper analysis of ITSS characteristics like different vascular abnormalities or iron deposition. Machine learning as well as deep learning algorithms can process this data to identify complex patterns, classify tumor subtypes, and predict aggressiveness or response to treatment with high accuracy. Together, radiomics and AI offer a powerful, non-invasive approach to improve diagnostic precision, facilitate personalized treatment planning, and advance our understanding of tumor heterogeneity (65–67).

## 5 Conclusion

ITSS can be seamlessly integrated into standard MRI protocols for both pre- and post-treatment evaluation of gliomas, providing a distinctive insight into tumor biology that correlates with key pathological features and clinical outcomes. It serves as a valuable biomarker, complementing findings from conventional and advanced neuroimaging techniques.

ITSS supports glioma differential diagnosis, grading, subtype differentiation, and treatment management, thereby contributing to precision and personalized medicine. Although challenges remain—particularly related to technical limitations and the lack of a unified model for ITSSs quantification—ongoing advancements in imaging technology and computational analysis, as well as longitudinal studies in this field, are expected to fully unlock the potential of ITSS in glioma management. Although the sensitivity in identifying ITSS increases with magnetic field intensity, the possibility of identifying such findings even with MRI scanners routinely used in clinical settings operating at 1.5 T, makes this imaging biomarker potentially increasingly important during neuroradiological evaluations and it is hoped that future studies on larger samples may further define the role of ITSS in the differential diagnosis, grading, and molecular profiling of brain gliomas.
